# Supplementary material for: Are carbon emissions trading and green financial instruments synergistic? -Comprehensive quantitative research based on content analysis
Source: PLoS One. 2024 Mar 7;19(3):e0298601. doi: 10.1371/journal.pone.0298601 (PMC10919601; doi:10.1371/journal.pone.0298601)
Supplement: S1 Data — (ZIP) [file pone.0298601.s001.zip › Data/地级市Z_F工作报告.zip!/╕╩╦α/░╫╥°╩╨/└┤╫╘╣1⁄2╓┌║┼í╛┬φ┐╦╩2╛▌═°í┐_2017.docx]

各位代表：

现在，我代表市人民政府向大会作工作报告，请予审议，并请政协委员和列席人员提出意见。

过去五年工作回顾

市八届人大一次会议以来的五年，是我市发展进程中不平凡的五年。在省委、省政府和市委的正确领导下，市政府全面贯彻党的十八大和十八届三中、四中、五中、六中全会精神，主动适应经济发展新常态，统筹推进稳增长、调结构、促改革、惠民生各项工作，较好地完成了“十二五”规划确定的目标任务，并实现了“十三五”的良好开局。

五年来，我们坚持把发展作为第一要务，全力以赴稳增长，综合实力稳步提升。按照稳中求进工作总基调，认真落实国家、省上促投资稳增长各项政策，不失时机制定并实施了促进非公经济、生产性服务业、现代物流业、文化体育旅游业、房地产业发展等一系列具体措施，实现经济平稳增长。累计完成固定资产投资2048.9亿元，是上个五年的2.7倍。实施亿元以上项目530个，完成投资932.7亿元。民间投资活力持续增强，新增市场主体3.8万户、注册资本881亿元，非公经济增加值占生产总值比重达到49.8%。获批国家循环经济示范城市、信息消费试点城市、信息惠民试点城市、首批产业废物综合利用示范基地和兰白承接产业转移示范区，争取国家、省级政策性资金478亿元。全市生产总值年均增长10%，固定资产投资年均增长18%，社会消费品零售总额年均增长10%，一般公共预算收入年均增长9.6%，城镇居民人均可支配收入年均增长10.9%，农村居民人均可支配收入年均增长13.3%。

五年来，我们坚持把产业转型作为主攻方向，坚定不移调结构，发展质量和效益逐步提高。在传统产业面临困境的情况下，审时度势,迎难而上,大力推进产业结构优化升级，三次产业结构由2011年的11.18:57.43:31.39调整为14.18:39.77:46.05。加快工业优化升级。一手抓传统产业改造提升，一手抓战略性新兴产业培育，不断延伸“3+4”产业链，实施工业项目280个，新增工业产值297亿元，规模以上工业增加值年均增长11%。白银公司铜铝导体新型材料和贵金属综合回收利用、稀土公司高性能抛光粉、刘化25万吨硝基复合肥、白银热电联产、华电景泰靖远风力发电、中电国际上沙沃光伏发电等项目建成投运。5户企业入选全省战略性新兴产业整体攻坚战骨干企业。大力发展循环经济。推进循环经济示范城市建设，高新区循环化改造进展顺利，银西工业园和平川经济开发区两个省级园区循环化改造基本完成。建成循环经济项目209个，完成投资509亿元，淘汰煤炭、电解铝等落后产能395万吨，工业固体废物综合利用率达到75%。稳步发展现代农业。实施“12313”现代农业培育工程，瓜菜、林果、草畜等优势产业加快发展，“三品一标”认证面积达到234.4万亩，新增农业龙头企业75家、农民专业合作社6510个、家庭农场524家，农产品加工转化率由49%提高到53%，规模化养殖比重由69.8%提高到79.2%。实施农产品出省工程，近三年累计向南方省份和京津冀等地销售农产品320万吨。建成省级现代农业示范区3个、示范园5个，白银国家农业科技园区获科技部批准建设。靖远县被确定为全国农村一二三产业融合发展试点示范县，平川区进入国家综合农业开发县区。实施土地整治项目159个，新增耕地2940公顷，建设高标准基本农田71.4万亩，耕地保有量和基本农田保护目标全面实现。粮食总产量稳定在75万吨以上。加速发展现代服务业。文化产业增加值年均增长27%，旅游业总收入年均增长25%。银东公铁联运和银西鼎丰、中进、邦农等物流重点项目建成投运。新改建农贸市场27个。建成县区电子商务中心5个、乡级服务站64个、村级服务点272个，电商企业达到200家，电子商务交易额突破2亿元，会宁、靖远、景泰列入国家电子商务进农村综合示范县。全市服务业增加值年均增长10%。

五年来，我们坚持把基础设施建设作为重要支撑，千方百计补短板，发展环境不断优化。实施“6873”交通突破行动、“6363”水利保障行动和生态保障工程，一批有利于长远发展、有利于民生改善的基础设施项目落地见效。全力破解交通瓶颈制约。完成交通基础建设投资88亿元。公路通车总里程达到1.22万公里，比2011年增加0.26万公里，建制村公路通畅率达到100%。营双高速景泰段、白银至桦皮川、吴家川至坝滩、黄河石林景区二级公路等建成通车。不断改善水利基础条件。完成水利基础建设投资38.8亿元。双永供水、兴电灌区齐家大岘隧洞改扩建、引大入秦延伸景泰供水工程建成投运，靖会甘沟干渠改扩建、引洮一期会宁北部供水工程完成主体建设。统筹推进城乡基础建设。投资45.86亿元，改善中心城区基础设施条件。新建城市道路27.7公里，维修改造25.3公里，城市生活垃圾无害化处理率、污水处理率、供水普及率、燃气普及率、绿化覆盖率、供热能力大幅提高。城镇化率由40.5%提高到47.8%。创建省市级美丽乡村41个，建成环境整洁村414个，改造农村危房6.3万户。持续改善生态环境。投资55亿元，实施矿山环境、工业污染源、重金属污染、东大沟流域河道等生态治理工程130项，黄河白银段和饮用水源地水质达标率均达100%。造林绿化159.3万亩，森林覆盖率由13.6%提高到14.3%，草原覆盖率由32.8%提高到41.8%。着力加强园区建设。白银工业集中区“一区六园”总规划面积340平方公里，累计开发土地91.7平方公里，入园企业465家，发展格局初步形成，成为转型发展的重要载体。

五年来，我们坚持把创新驱动作为第一动力，持之以恒促改革，发展活力有效激发。用改革的办法、创新的思路破解发展难题，促进经济转型升级。围绕重点领域推进改革。完成“四单一网”建设，取消、调整、下放行政审批事项577项。深化国有企业改革，积极承接白银公司、银光公司、靖煤集团、稀土公司等企业办社会职能，妥善解决13户改制企业欠缴养老金等历史遗留问题。加快农村产权制度和林权改革，流转土地122.3万亩。推进金融体制改革，菁茂农业、丝路林牧等231家企业在“新三板”和区域性股权交易中心挂牌，直接融资87亿元。商事制度、户籍制度、教育、医疗卫生、供销社等重点领域改革扎实推进。围绕兰白科技创新改革试验区建设促进创新。建成各级工程技术研究中心34家、省级重点实验室2个，取得科技创新成果283项，培育高新技术企业26家、科技型企业91家，组建战略创新联盟6个，中科院近代物理研究所、化学物理研究所在我市设立中试基地。兰州大学白银产业技术研究院、兰州理工大学白银新材料研究院建成运营。科技企业孵化器被认定为国家级孵化器、首批国家小微企业创业创新示范基地，白银创新梦工厂被认定为国家级众创空间。每万人发明专利拥有量达到1.6件，科技进步贡献率由2011年的47%提高到52%。围绕丝绸之路经济带建设扩大开放。加强与丝绸之路经济带沿线地区交流合作，支持白银公司等骨干企业成功实施国际产能合作项目，鼎丰保税物流仓封关运营，进出口总额年均增长8.1%。利用兰洽会、津洽会、广交会、文博会等各类招商引资平台，签约招商引资项目864个，完成投资1599.6亿元。

五年来，我们坚持把全面小康作为根本目标，尽心竭力惠民生，群众生活持续改善。顺应广大群众新期待，推进社会建设和民生改善，累计用于民生方面投入473亿元，是上个五年的2.3倍。扎实推进脱贫攻坚。实施“1236”扶贫攻坚工程和“双联”行动，投入专项扶贫资金11.3亿元，发放各类扶贫惠农贷款99.2亿元，改善基础设施条件，提升公共服务水平，培育富民多元产业，拓宽贫困群众增收渠道，农村贫困人口由54.8万人减少到23.3万人，贫困发生率由41%下降到17.3%。坚持就业优先政策导向。落实就业专项资金6.1亿元，开展职业技能、岗位技能和创业培训30.4万人，新增城镇就业30.3万人，输转城乡富余劳动力148.8万人次，创劳务收入210.4亿元，城镇登记失业率控制在4%以内。完善城乡社会保障体系。城乡居民基本养老保险参保率达到95.6%，企业退休人员养老金、城乡居民基础养老金分别提高794元和30元，新农合和城镇居民医保人均政府补助标准均提高220元。建设保障性安居工程4.5万套，白银城区改造旧住宅区180.4万平方米。大力发展社会事业。新改扩建校舍166.2万平方米，全面消除中小学D级危房，乡镇中心幼儿园和2000人以上行政村幼儿园实现全覆盖，九年义务教育巩固率达到99.4%，高中阶段毛入学率达到92.3%，公民平均受教育年限达到10.6年,景泰县、白银区、平川区通过国家义务教育基本均衡县评估认定。成立白银矿冶职业技术学院，填补了我市高职院校的空白。文化体育基础设施不断完善，文化馆、博物馆、图书馆免费开放，新建“一村一场”体育工程536个。市第一人民医院门诊楼、市儿童医院、省中医院制剂中心、县区人民医院住院部等一批医疗基础设施建成运行，标准化村卫生室实现全覆盖。“全面两孩”政策有序实施。重视加强社会治理。落实“六五”普法规划，加强社会治安综合治理,推进“平安白银”建设,建立城市大巡防体系,命案和八类主要刑事案件发案率分别下降71%、69%。安全生产和食品药品监管持续加强,信访维稳工作扎实有效，第四次荣膺全国双拥模范城市，获得全国文明城市提名资格。

五年来，我们坚持把提升效能作为主要抓手，下大气力转作风，政府自身建设进一步加强。在狠抓经济社会发展的同时,突出为民、务实、清廉,加强学习型、创新型、法治型、服务型、责任型、廉洁型政府建设。扎实开展党的群众路线教育实践活动和“三严三实”、“两学一做”学习教育，大力推进政府职能转变，坚决整治不作为、乱作为和庸懒散慢等“四风”问题。自觉接受人大依法监督和政协民主监督，累计办理人大代表建议406件、政协委员提案855件。认真落实省委“3783”、市委“755”党风廉政建设主体责任,严格执行中央八项规定、省委“双十条”和市委“十六项”规定，政府系统党风廉政建设取得明显成效。从严控制部门预算，削减压缩一般性支出，“三公”经费下降52%。深入开展专项检查和审计监督，严肃处理违纪违规问题，问责主体责任落实不到位单位31个、领导干部77名,政风行风持续好转。

与此同时，外事侨务、防震减灾、统计、气象、地方志、档案、老龄、妇女儿童、残疾人、红十字等工作取得新成绩，民族团结、宗教和顺的局面更加巩固。

各位代表，即将过去的2016年，是本届政府任期的最后一年。在多重困难和挑战压力下，我们积极作为，有效应对，按照市委的总体部署和“十三五”规划纲要，突出抓了事关白银转型发展的基础性、关键性工作。

一是突出重点抓扶贫、促脱贫。整合各类资金34.8亿元，发放专项贷款14.8亿元，建成贫困村通畅工程328.9公里，新改建集中供水工程24处，改造农村危房1.2万户，改扩建贫困村幼儿园78个，开工建设易地扶贫搬迁工程安置点29个。在贫困地区发展瓜菜3.4万亩，新建规模养殖场29个，输转贫困户富余劳动力5.6万人，创劳务收入5.2亿元。预计7.15万贫困人口实现脱贫。

二是多措并举抓项目、促投资。实施各类项目1166个，其中新开工844个，完成投资342亿元，同比增长18%左右。中核钛白20万吨钛白粉一期土建工程即将完工，昌元化工10万吨重铬酸钠一期加紧实施，华鹭铝业出城入园项目开工建设，白银公司锌冶炼资源综合利用项目投入试运行，国道247线靖远至会宁侯川段改扩建、省道217线景泰大水石奓至白银二级公路改扩建等重大基础设施建设项目加快推进，黄河白银段防洪工程第一批项目各标段基本完成，地下综合管廊试点项目完成投资10.4亿元，包兰铁路中卫至兰州客运专线开工在即，国际青少年儿童美术博览馆完成主体工程。

三是坚持不懈抓改革、促创新。简化优化服务流程，市级行政审批服务事项实现流程再造,启动运行投资项目在线审批监管平台，推行集中审批、网上审批、并联审批。完成市级公务用车改革。推进供给侧结构性改革，淘汰电解铝落后产能23万吨、煤炭产能87万吨，消化商品房库存41万平方米。推进兰白科技创新改革试验区建设，与张江自主创新示范区、上海产业技术研究院、兰州大学等签订合作协议11项，设立总规模9亿元的科技创新创业投资基金和大健康产业发展基金，科技企业孵化器新入孵企业30家，入驻创业团队72个，成立众创空间14家。

四是不遗余力抓民生、促和谐。全市财政用于民生支出同比增长12.2%，占总支出的83%。省市确定的20件惠民实事全部完成。新增城镇就业5.7万人，实施棚户区改造7333户，农村低保一二类标准和扶贫脱贫线“两线合一”，免除学前教育幼儿保教费和建档立卡贫困家庭高中生学杂费、书本费，公立医院综合改革和分级诊疗、医师多点执业等医药卫生体制改革重点工作深入推进。

经过全市上下的共同努力，预计2016年实现生产总值430亿元，同比增长6.5%；规模以上工业增加值110亿元，增长5%；固定资产投资526亿元，增长11%；社会消费品零售总额190亿元，增长8%；一般公共预算收入27亿元，增长6%；城镇居民人均可支配收入25313元，增长8%；农村居民人均可支配收入7772元，增长10%。

各位代表！过去的五年，我们万众一心，开拓进取，在团结奋进中凝聚力量，在攻坚克难中砥砺前行，全市经济社会发展取得了前所未有的新成就。这是省委、省政府和市委正确领导的结果，是全市人民同心同德、奋力拼搏的结果。在此，我代表市政府，向全市广大工人、农民、知识分子、各级干部和历任老领导、老同志，向各民主党派、工商联、无党派和社会各界人士，向驻银部队、武警官兵、公安干警和中央、省驻银单位，向所有关心、支持白银发展的各界朋友，致以崇高的敬意和衷心的感谢！

五年来的实践启示我们,在新的起点上推进白银经济社会发展，必须始终坚持党的领导,突出责任担当,不折不扣地贯彻落实中央和省委、市委的方针政策，确保国家和省市重大决策在白银落地生根；必须始终坚持转型发展,突出工业强市,注重发展循环经济，提升发展层次和质量；必须始终坚持改革创新,突出问题导向,向创新要动力、向改革要活力，为加快发展增添后劲；必须始终坚持为民宗旨,突出脱贫攻坚,切实改善群众生活，使人民群众共享发展成果；必须始终坚持求真务实,突出改进作风,进一步加强政府自身建设，提高履职尽责能力和水平。这些经验是全市人民智慧的结晶,弥足珍贵,我们一定要倍加珍惜、发扬光大。

在看到成绩的同时，我们也清醒地认识到，我市正处在滚石上山、爬坡过坎的关键时期，发展过程中还存在不少矛盾和问题，前进道路上还面临许多困难和挑战。经济总量小，产业层次低，总体实力弱，继续保持中高速发展的难度和压力较大；大项目、好项目储备少，产业性重大项目接续不足，新旧动能转换任重道远；企业创新主体地位不够凸显，创新人才严重短缺，科技创新整体竞争力不强；农业持续增效、农民持续增收难度加大，脱贫攻坚任务艰巨；主导产业效益下滑，骨干财源培植不足，财政收支矛盾突出；一些干部思想解放程度不够，担当精神不足，谋划发展的办法不多，推动工作的能力不强，致使既有的政策平台和资源能源等优势没有充分转化为现实生产力。我们必须以对党、对人民高度负责的精神，正视问题，直面挑战，采取有效措施，在今后的工作中认真加以解决。

今后五年主要目标和重点任务

未来五年，是我市全面建成小康社会的决胜期，也是经济社会转型发展的关键期。作为老工业基地、资源型城市和欠发达地区，发展不足是我们的主要矛盾，脱贫攻坚是我们的头等大事，转型发展是我们的根本任务。虽然我们面临发展大环境不确定因素增多、经济下行压力持续加大、区域竞争更趋激烈的严峻复杂形势，但全市发展总体仍处于可以大有作为的重要战略机遇期。国家“一带一路”、新一轮西部大开发、老工业基地振兴、脱贫攻坚等战略的实施，以及资源型城市转型、大兰州经济区、兰白科技创新改革试验区建设等政策优势的进一步释放，将对我市经济发展起到重要的拉动作用。多年来我市在转型升级、“3+4”产业集群、园区建设、科技创新等方面打下的坚实基础，正在形成新的比较优势，并将逐步转化为发展优势。面对内部条件与外部环境交互影响、短期因素与长期因素耦合作用、上行推力与下行压力博弈对冲，我们要以全市“十三五”发展战略目标为标尺，用全面、客观、辩证、发展的眼光，正确认识、科学把握我市经济社会发展的基本面和发展趋势，树立发展信心不懈怠，保持发展定力不动摇，咬定发展目标不放松，科学谋划，主动作为，努力促进我市经济社会全面转型升级。

刚刚闭幕的市第八次党代会，明确提出了我市今后发展的总体要求，这就是：高举中国特色社会主义伟大旗帜，以马克思列宁主义、毛泽东思想、邓小平理论、“三个代表”重要思想和科学发展观为指导，全面贯彻党的十八大和十八届三中、四中、五中、六中全会精神，深入贯彻习近平总书记系列重要讲话精神，统筹推进“五位一体”总体布局，协调推进“四个全面”战略布局，牢固树立五大发展理念，认真落实“八个着力”重要指示，围绕实现与全国全省同步进入全面小康社会的宏伟目标，更加注重创新驱动、更加注重转型升级、更加注重开放开发、更加注重改善民生、更加注重党的建设，努力建设繁荣文明幸福新白银。

今后五年的奋斗目标是：生产总值年均增长8%，一般公共预算收入与经济保持同步增速，固定资产投资年均增长12%，社会消费品零售总额年均增长8%，居民消费价格指数控制在103以内，城镇居民人均可支配收入年均增长7.5%，农村居民人均可支配收入年均增长9.5%。经过五年的努力，全市经济社会发展实现“两翻番、两倍增、两确保”，即地区生产总值和城乡居民人均可支配收入比2010年翻一番以上；一般公共预算收入和固定资产投资在2015年基础上实现倍增；确保非公经济比重达到55%以上、城镇化率达到53%以上。

围绕以上总体要求和发展目标，今后五年政府工作的着力重点是：

一、实施“四大工程”，着力厚植发展实力，建设优势鲜明的新型工业城市。深入实施工业强市战略，推动“3+4”产业集群更快、更优发展，大力发展现代服务业，形成传统产业与新兴产业“双轮驱动”、新型工业与现代服务业“双业并进”的发展态势，构建现代产业新体系。

实施传统产业改造提升工程。传统产业是我市经济增长的“稳定器”。要充分运用老工业基地调整改造政策，推动传统产业与互联网融合发展，开展“两化”融合试点示范，打开传统产业成长新空间，重塑传统产业竞争新优势。依托技术创新，延伸铜基、铝基、锌基、贵金属等产业链，使铜铅锌冶炼工艺技术达到国际先进水平，推进锦江集团铝产业链项目，建设多品种有色金属工业基地。延伸以TDI为核心的光气化产业链，打造全国最大的含能材料生产研发及聚氨酯产业园；推动传统氟化工向精细氟化工、电子氟化工转变，打造氟化工产业园，建设西部重要的新型化工基地。实施一批新能源项目，建设西部综合能源开发转化消纳基地。加快发展中高端陶瓷制品、新型墙体材料，推动陶瓷产业向节能、绿色、精细化转变，建设西部陶瓷建材产业基地。大力发展循环经济，投资450亿元，实施冶炼渣综合利用、新型节能环保建材生产线、废旧汽车回收拆解利用、餐厨废弃物处理、固废垃圾无害化处理等120个循环经济重点项目，基本构建围绕主导产业发展的循环经济产业集群和生态产业链条，建设高标准国家级循环经济示范市和资源型城市转型明星城市。

实施新兴产业培育壮大工程。战略性新兴产业是我市结构调整的“加速器”。要全力推动生物医药、碳纤维材料、新能源装备制造、稀土新材料产业园建设，培育一批具有区域影响力的创新型企业。以有色金属、稀土功能、碳纤维、凹凸棒石等材料为重点，建设西部有色金属新材料基地。实施新型煤化工示范项目，促进煤炭资源就地转化增值和循环利用。积极发展电气机械及器材、大型采矿设备、自动化控制设备等装备制造业，逐步实现规模化、高端化。加快发展生物医药、节能环保、电子信息等技术密集型产业，推动产业由资源主导型向科技主导型转变。战略性新兴产业增加值占生产总值比重提高到20%。

实施现代服务业扩量提质工程。现代服务业是我市转型升级的“助推器”。要根据我市的资源禀赋和产业基础，利用产业政策的导向功能，推动生产性、生活性服务业发展提速、比重提升、水平提高。大力发展现代物流、电子商务、金融服务等生产性服务业,构建“一个枢纽、两大基地、五个聚集区”现代物流产业格局,建设电子商务创业园区和区域金融中心。加快发展健康、养老、家政、文化旅游、体育休闲、信息消费等生活性服务业,构建医养融合健康服务体系，打造陇中养老服务产业核心区，加快黄河石林、会师旧址“两大精品景区”建设,推进景泰县国家全域旅游示范县建设,建设市体育文化产业园，促进传统生活消费方式向现代型、服务型转变。

实施工业园区提档升级工程。工业园区是我市产业集聚的“承载器”。要以白银高新区为龙头，加快银东、银西、刘川、平川、正路、会宁等园区基础设施建设，提升园区承载能力。优化园区体制机制，把“一区六园”打造成产业集聚平台、招商引资平台、创新创业平台、公共服务平台和人才引育平台。围绕“3+4”产业链，合理布局“一区六园”产业，促进产业集群化发展。到2021年，“一区六园”累计完成固定资产投资1130亿元，工业产值达到900亿元。

二、突出“两个引领”，着力激发发展动能，建设活力迸发的创新创业城市。充分发挥改革创新的引领作用，以兰白科技创新改革试验区建设为统领，强力推动科技创新，以供给侧结构性改革为重点，统筹推进改革开放。

突出创新引领，为发展注入新动力。实施创新驱动战略，把兰白试验区作为创新发展的战略支点，认真落实兰白试验区建设规划，加快实施“六个一百”工程，切实在创新体系建设、科技成果转化、体制机制改革等方面取得新的突破。构建创新体系。建成国家级重点实验室和企业技术中心各1家、省级工程技术研究中心10家，培育高新技术企业40家、科技型企业200家。加快科技成果转化。建成科技企业孵化器8家、国家级孵化器2家，在孵企业达到1000家，建设市技术大市场，创建国家知识产权示范城市，专利申请量年均增长20%以上，万人发明专利拥有量达到4.5件，全社会研发投入占生产总值的比重达到2.5%，科技进步贡献率达到55%。完善鼓励创新创业体制机制。大力培育创新创业主体，推动企业“双创”平台建设，创建银西产业园省级“双创”示范基地，建成省级创业就业孵化示范基地（园区）10个。加大创新创业金融支持，投放创业担保贷款15亿元以上，筹措担保基金9000万元。重视创新创业人才引育，加强本土技能型、实用型人才培养，实施急需紧缺高层次人才柔性引进计划，综合施策，激励各类人才创新创业。

突出改革引领，为发展增添新活力。抓住制约发展的重点领域和关键环节推进改革，以改革增强发展活力、推动持续发展。扎实推进供给侧结构性改革。坚决停止审批新增煤炭等落后过剩产能项目，淘汰煤炭落后产能54万吨。通过棚改货币化安置、购租并举、释放农民工住房需求等措施，三年内消化存量商品住房127.2万平方米，促进房地产业健康发展。降低企业“过桥”融资成本，扩大直接融资规模，力争实现直接融资200亿元以上。进一步减轻企业税费等负担，降低企业制度性交易、人工、用能用地和物流成本，推动企业分离办社会职能。加大对薄弱环节、重点领域投入力度，积极争取补短板重大项目，全面落实补短板重点任务。持续深化重点领域改革。继续推进“放管服”改革，进一步简化优化行政审批流程。深化国有企业股份制改革，完善法人治理结构，发展混合所有制经济，放大国有资本功能。支持非公经济健康发展，优化要素供给的体制机制，激发非公经济发展动力和活力，形成一批跨地区、跨行业、跨所有制、具有核心竞争力的大型民营企业集团。推进电力体制改革，加快推进电网智能化建设。深化农村土地“三权分置”、农村集体经营性资产、农村金融、供销社等领域改革，盘活农村“沉睡”资源资产。深化公共服务提供方式改革，大力推行政府购买服务。不断提高对外开放水平。积极承接东中部产业转移项目，建设兰白承接产业转移示范区。以有色、化工等优势产业国际合作为先导，带动中医药、文化、旅游等特色服务贸易进入国际市场，建设丝绸之路经济带国际产能合作区。

三、聚焦“四大任务”，着力促进发展成果惠及人民，建设普惠共享的全面小康城市。坚持民生优先理念，聚焦脱贫攻坚、农业产业化、基础设施建设、公共服务供给精准发力，改善群众生产生活条件，增进群众福祉。

坚决打赢脱贫攻坚战。把全面小康、脱贫攻坚作为全局工作的重中之重，以“六个精准”为主要抓手，进一步落实“1+18”精准扶贫方案，全力抓好脱贫攻坚“一号工程”。做细做实“853”精准脱贫管理，建立贫困户收入状况精准监测和脱贫认定机制。加大扶贫资金投入力度，建立县级扶贫开发资金项目整合管理平台。继续推进专项扶贫、行业扶贫和社会扶贫，实施发展生产、转移就业、易地搬迁、生态补偿、教育扶贫、医疗救助及社会保障兜底“七个一批”扶贫工程，完善贫困村“水、电、路、房”等基础设施。每年稳定减少贫困人口5万人以上，到2020年，稳定实现农村贫困人口不愁吃、不愁穿，义务教育、基本医疗和住房安全有保障，扶贫对象人均可支配收入增幅高于全市平均水平，基本公共服务主要领域指标接近全国平均水平，现行标准下农村贫困人口实现脱贫，贫困县区全部摘帽，实现区域整体脱贫。

扎实推进农业产业化。把农业产业化作为农民增收的根本途径，大力发展瓜菜、草畜、优质林果、粮油四大主导产业和马铃薯、中药材、小杂粮、乡村旅游四大特色产业。以沿黄灌区为主，发展设施养殖、优质瓜菜、现代制种等产业，建设高效农业示范带；以高扬程灌区为主，发展节水种植、草食畜牧、特色林果等产业，建设立体复合型农业经济区；以南部旱作区为主,发展马铃薯、小杂粮、玉米、饲草等产业，建设旱作特色农业经济区。以白银国家农业科技园区为依托，以农产品加工企业为龙头，以县区为主体，集中力量建设一批标准化、规模化、集约化的现代农业产业基地，重点建设标准化规模种植基地30个、养殖场（小区）300个，新增农业龙头企业50家，农产品加工转化率提高到60%，推动农村一二三产业融合发展，提高农业竞争力，持续、稳定增加农民收入。

大力改善基础设施条件。紧紧抓住国家加快西部地区基础设施建设的机遇，加快推进以交通水利为重点的基础设施建设。新建高速公路152公里,新改建国省干线公路1173公里、各类农村公路2000公里，提高公路网密度。配合实施包兰铁路中卫至兰州客运专线、干武铁路增建二线工程，推进环海中、白银至平凉、环兰州城际会宁至平川段等铁路连通工程，构建“三横三纵”铁路交通网络。推进引洮会宁供水、新一轮大中型灌区续建配套与节水改造等骨干水利工程建设，实施兴电灌区维修改造、甘肃中部生态移民扶贫开发供水、景电三期景泰片区电力提灌等工程，发展耕地灌溉面积10万亩，建设全省生态移民示范区。

努力提高基本公共服务水平。以改善民生福祉、让人民群众享有公平教育、优质医疗、丰富文化、完善社保和可靠的公共安全为目标，全面加快基本公共服务均等化。为群众提供更公平的教育资源。大力发展学前教育，实现60%以上的乡镇中心幼儿园标准化办园。改善义务教育薄弱学校条件，2018年实现县域义务教育基本均衡。促进普通高中优质、特色、多样化发展，提升白银矿冶职业技术学院和全市中等职业教育水平，推动兰州城市学院和靖远师范合作办学。为群众提供更优质的医疗服务。加快健康白银建设，推进医改向纵深发展，提高市县公立医院医疗水平，鼓励发展社会办医，做强基层医疗服务，做实基本公共卫生和优生优育服务。为群众提供更丰富的文化服务。建成市文化艺术中心、国家矿山公园、3D数字博物馆、数字群众艺术馆，推进县区图书馆、文化馆达标，改造提升乡镇综合文化站，实现市、县、乡、村四级公共文化服务网络全覆盖。为群众提供更完善的社会保障。实施全民参保登记计划，巩固低保“应保尽保”成果，稳步推进收入分配制度改革，持续提高城乡居民收入水平。采取劳动者自主就业、市场调节就业和政府促进就业等多种途径，推动更加积极的就业政策落实。在提高基本公共服务水平的同时，重视社会治安综合治理、安全生产、食品药品监管等工作，有效防范、化解、管控各类风险，为群众提供良好的公共安全保障。

四、推进“三项建设”，着力优化发展环境，建设富有魅力的宜居宜业城市。推进新型城镇、生态环境、智慧城市建设，改善城乡面貌，提升发展内涵。

建设设施先进、功能完善的新型城镇。积极融入以兰白都市圈为核心的中部城市群建设，统筹推进规划、建设、管理，大力提高城镇化水平。推进中心城区建设和管理。实施一批城市基础设施建设项目，建设中心城区道路19条38.2公里、地下综合管廊34.8公里，改造供水干网12.1公里、住宅小区供水管网99.5公里，实现热电联产供热全覆盖。加快老旧城区、“城中村”改造，完成棚户区改造2.5万户。推进大环境绿化，提高绿化覆盖率。生活垃圾无害化处理率、城市污水处理率、城市供水普及率均达到100%。进一步理顺城市管理体制，推动城市管理重心下移。探索城市管理运营新模式，促进城市管理人性化、智能化、精细化。推进白银区、平川区和刘川新区一体化发展，构建大白银城市格局。加快县城和小城镇建设。以提质扩容和产业支撑为重点，着力打造“四条经济带”，以产业带动就业、集聚人口，以产业繁荣城镇，创建省级产城融合示范区。加快城镇“三供两处两场”基础设施建设，从实际出发逐步实施县城地下管廊工程。抓好特色小城镇建设，打造区域新型城镇化标杆。

建设山川秀美、绿色环保的生态环境。抢抓国家生态安全屏障综合试验区建设机遇，以实施重大生态建设工程为主导,努力改善生态环境。筑牢生态屏障使山川更绿。推进黄河上游白银段生态环境综合治理，实施生态系统保护与恢复、环境综合治理、水土资源优化配置与生态移民、生态产业扶持“四项工程”，构筑以“两屏夹一带”为主体的西部重要生态安全屏障，森林覆盖率达到14.7%。提高空气质量使天空更蓝。做好政策、规划和项目环评工作，推行环境污染第三方治理。用铁的手腕、硬的措施，打好多污染物协同控制攻坚战，到2021年，主要污染物排放总量显著减少，空气质量二级以上优良天数占比达到85%以上。强化土地整治使大地更洁。加强土地、矿产等资源的节约循环利用，加大采煤沉陷区、盐碱地和重点流域综合治理，新增水土流失治理面积600平方公里，新修梯田44万亩。开展农业面源污染治理，有效遏制土壤污染。加强重金属污染治理修复和矿山地质环境恢复治理，在“多还旧账”的基础上“不欠新账”。

建设深度互联、协同共享的智慧城市。以“互联网+”为主题，以国家“智慧城市”、“信息惠民”、“信息消费”试点城市建设为抓手，聚焦城市管理、政务服务、民生服务、产业发展、绿色节能等重点领域，建成“一中心一平台十一大应用”智慧城市系统，实现城市智慧式管理和运行。重点推进政务服务事项“一号”申请、“一窗”受理、“一网”通办，实现行政许可、行政处罚事项90%以上通过网络办理，政务信息95%以上通过网上发布，城市网格化管理达到95%，70%以上的市民通过网络享受交通、医疗、教育、文化和社会保障等领域智慧应用服务，使智慧城市建设成果惠及全体市民。

2017年重点工作

2017年，是新一届政府履行职责的开局之年，也是实施“十三五”规划、推进全面小康的关键之年。政府工作要按照中央、省上的总体部署和市第八次党代会精神，聚焦聚力，抓主抓重，努力实现全市经济社会发展各项目标任务。

主要预期目标是：生产总值增长7.5％，规模以上工业增加值增长6.2%，固定资产投资增长12％，社会消费品零售总额增长8％，一般公共预算收入增长8％，城镇居民人均可支配收入增长8％，农村居民人均可支配收入增长9.5％，居民消费价格总水平涨幅控制在3％以内。

为实现上述目标，重点抓好以下工作：

一、以项目建设为支撑，确保有效投资较快增长

抓项目建设，扩大投资增量。完成国道109线白银至兰州段、国道247线靖远至会宁侯川段建设，加快国道338线景泰至年家井段、省道217线景泰大水石奓至白银等项目建设进度，全面开工建设景泰至中川高速、国道341线白银至中川机场一级公路、省道315线景泰段、省道209线平川段。继续抓好白银公司低品位含锌杂料综合回收示范、稀土公司高纯稀土金属及特种合金生产线、锦江集团铝产业链、华鹭铝业出城入园、恒大年产2450万平方米陶瓷砖生产线、昌元化工10万吨重铬酸钠二期、中天化工2000吨六氟磷酸锂、靖远年产6000吨电子级及太阳能级多晶硅等工业项目建设。实施基本建设项目865个，力争完成年度投资592亿元。

抓政策机遇，挖掘投资潜力。瞄准国家“11+6+3+1”重大工程建设布局，对接国家、省“十三五”规划及各专项规划，凝炼、生成、储备一批投资强度大、科技含量高、资源消耗低、市场前景广的优质项目。做好靖煤年产21万吨煤制乙二醇、PX石油化工等项目和已列入国家、省市规划项目的前期工作，创造条件尽快开工建设。创新招商引资机制体制，提高签约项目质量，到位资金达到545亿元以上。

抓融资平台，拓宽投资渠道。强化银行对地方经济发展的信贷支持，引导保险资金投资支持重点项目建设。加大企业多层次资本市场融资力度，鼓励引导符合条件的企业发行专项债券，创造条件发展融资租赁业务。探索建立水权流转交易市场，积极争取省级平台政府性债务置换额度。建立和完善融资担保、信用保证、风险补偿、基金引导、股权驱动等中小企业信贷支持机制，规范发展小额贷款公司和融资性担保公司，试点推进小额贷款保证保险，为“三农”、小微企业提供更多融资支持。全面完成农村土地确权登记颁证，建立农村产权流转交易平台，推进农村生产要素合理配置。

抓非公经济，优化投资结构。全面落实国务院和省政府促进创业投资和民营企业发展政策措施，放宽非公经济准入条件，搭建服务平台，促进非公经济“小升规”、“个转企”，新增规模以上工业企业25户。加快实施29个已开工的PPP项目，尽快开工已开展前期工作的项目，面向社会推出一批新的PPP项目，争取更多项目入库落地。民间投资占全部投资比重保持在50%以上。

二、以精准脱贫为核心，促进农村经济全面发展

抓实抓细，推进精准脱贫。实施易地搬迁1.09万户、4.56万人，解决5万农村人口安全饮水问题。开展农民技术培训10万人次，输转农村富余劳动力28万人次。整合扶贫资金41亿元，重点用于基础设施建设、富民产业培育。推行光伏扶贫、电商扶贫、健康扶贫等新型扶贫模式。继续推进农村低保一、二类对象和特困供养人员“政策性脱贫”，建立多层次、全覆盖的贫困家庭在校生助学体系。减少贫困人口8.25万人，景泰县脱贫摘帽。

转变方式，提高农业效益。继续调整种植结构，全面推广全膜玉米和黑膜马铃薯覆盖栽培技术，培育富民特色产业，新增蔬菜种植3.3万亩、中药材1万亩，新增标准化规模养殖场（小区）50个。新增农业龙头企业10户,农产品加工转化率达到54%。开展国家级、省级农产品质量安全县（区）创建工作,加强“三品一标”农产品认证,确保蔬菜、畜禽和水产品平均合格率均达到98%以上。推进白银国家农业科技园区建设，大力发展品牌农业,积极培育国家级、省级知名品牌。

加大力度，夯实发展基础。加快黄河干流白银段防洪治理、大型泵站更新改造、高效节水和小型农田水利项目建设，实施兴电灌区维修改造工程，推进甘肃中部生态移民扶贫开发供水、李家塬电力提灌等项目立项建设，发展耕地灌溉2万亩，完成水土流失治理120平方公里。新建通村公路620公里。完成25个美丽乡村示范村和138个环境整洁村建设任务，村庄生活垃圾彻底治理率达到70%以上。实施电信普遍服务试点项目，新建和改造升级484个行政村光纤宽带，实现所有行政村通宽带。

三、以破解结构性矛盾为关键，助推工业经济优化发展

优化结构，提升产业层次。围绕有色、化工、能源等传统支柱产业，实施重点技术改造项目，鼓励优势企业通过资本、技术等整合重组，实现集团化、集约化发展。支持白银公司加快“两化”融合，推进战略引资和整体股改上市，做好靖煤集团社保、医疗、教育等8个企业办社会机构移交后续工作，积极承接银光公司企业办社会职能。制定中国制造2025白银落实方案，对接全省战略性新兴产业总体攻坚战，促进战略性新兴产业上档次、扩规模,新增骨干企业2户，规模以上战略性新兴产业增加值占规模以上工业增加值比重提高1.5个百分点。

优化引擎，提升增长动能。全面推进兰白试验区建设，加强与上海张江等自主创新示范区和科研院所的合作，促进重大科技项目、高端研发团队、创新基地与服务平台的引进落地。瞄准市场需求，加快“创业苗圃+孵化器+加速器”孵化体系建设，新招孵企业50家。推进“双创”示范基地建设，新增省级众创空间10家以上，培育省级工程技术中心1个。瞄准结构优配，催生一批科技型企业,新增高新技术企业5家、科技型企业10家，培育2家科技型企业进入“小巨人”企业。瞄准产业风景，利用现有企业和技术优势，重点培育通用航空、超导、风电装备服务型制造、大健康、节能环保材料、动力电池制造等先导性产业集群。

优化载体，提升园区配套。加快园区供电、供水、道路、污水处理、管网、信息化等基础设施建设，重点实施好刘川工业园1亿美元亚行贷款项目，抓好银西产业园、平川经济开发区南区、景泰正路工业园基础设施建设，建成各工业园区集中式污水处理厂。推行“一站式”服务，提升园区综合服务能力。“一区六园”新入驻企业68家，完成投资179亿元，产值达到570亿元。

优化服务，提升要素保障。开展“企业服务年”活动，构建“发展同谋、改革同抓、城市同建”的新型地企协作关系。搭建政府部门与企业之间对话交流平台，实现沟通协调多层化、高效化、常态化。协同谋划地企发展，将大中型骨干企业的发展规划融入全市“十三五”规划和各专项规划，使地方和企业发展思路、产业规划无缝对接、高度契合。增强主动服务意识，在行政审批、项目申报、建设用地、煤电油运、股改上市等方面做好协调服务，为企业排忧解难。

四、以促进消费为导向，推动第三产业加快发展

推动文化旅游产业上水平。大力培育本土骨干文化旅游企业，加快丝绸之路文化遗产博览城暨黄河石林景区、黄河石林特色小镇建设,启动黄河石林5A级景区、会师旧址国家级红色旅游基地创建工作，打造智慧旅游城区、智慧景区、智慧旅行社、智慧旅游饭店。提升乡村文化旅游发展水平，开工建设水川黄河假日城湿地公园二期和“袁家村·白银印象”项目，新建专业旅游村3个、标准化农家乐25户。积极推进与兰州、临夏、中卫等沿黄市州旅游联盟建设，确保精品线路吸引游客突破80万人次。

推动商贸流通业扩规模。加快银西商贸、银东工业物流园等重点物流项目建设，新建商贸中心2个，新改扩建农贸市场6个。改造提升村级供销社经营服务网点，在中心村和人口密度大的行政村规范建设农村(社区)综合服务社。完善外贸促小育新项目库，争取全省小微外贸企业互助贷款担保基金及各项外经贸发展资金支持，促进外贸企业发展。全市进出口总额增长5%。

推动新兴业态快发展。打造农村电商、城市电商、跨境电商“三大板块”，实现全市70%以上的村建成电子商务服务点，电子商务交易额和网络零售额增长30%，物流快递到乡镇、功能覆盖到村。认真谋划实施一批新型消费项目，满足群众多样化、个性化消费需求。

五、以基础设施建设为重点，推进新型城镇化建设

完善基础设施。完成市区上海路北段、凤凰街、东纬二路等6条道路建设，新建银山路、北环路西段等5条道路，打通瓶颈路，畅通城市交通。加快推进白银城区防洪及综合治理、地下综合管廊试点项目，提升城市综合功能。强化县城规划设计，完善县城功能配套。加快推进“一县两镇”省级新型城镇化试点和1个省级、5个市级特色小镇建设，助推新型城镇化建设。

加强城市管理。推进城市综合执法改革，推广数字化城市管理模式。以全国文明城市创建为统揽，推进“五城联创”、“六城同建”，重塑老工业城市新形象。加强市容市貌整治，扩大交通“严管街”范围，建立应急处置机制，下决心解决群众关心的交通拥堵、管理粗放、应急滞后等问题。加快推进农业转移人口市民化，城镇化率提高1.3个百分点。

改善人居环境。完成高新区循环化改造，力争35户循环经济骨干企业进入国家和省上试点示范行列。提升资源综合利用水平，农作物秸秆资源综合利用率达到82%，废旧农膜回收利用率达到80%。推进生态林业建设，完成营造林面积32万亩。抓好有色、稀土及农副产品加工等重点行业水污染防治，实施东大沟河道重金属污染治理、历史遗留铬污染土壤治理和采煤沉陷区生态环境恢复治理工程，完成白银公司和稀土公司重金属污染治理项目。城市空气质量优良天数达到290天以上。靖远、景泰、平川热电联产项目和会宁县集中供热工程建成运营。

六、以改善民生为根本，保持社会和谐稳定

真情惠民，让公共服务更优质。实施好全面改薄、行政村幼儿园建设项目和乡村教师支持计划，会宁县通过国家义务教育基本均衡县评估认定。加强普通高中教育和继续教育、特殊教育工作，深化产教融合，推进教育协调发展。改善医疗卫生基础设施条件，加快市第一人民医院住院部、市妇幼保健院综合楼、市全科医生培训基地、会宁县人民医院建设，改扩建5个乡镇卫生院，推进公立医院综合改革，大力发展中医药事业。加强育龄人口生育、生殖保健服务，提升计划生育服务水平。继续开展全国第三批公共文化服务体系示范区创建工作，推进“文化集市”建设。加快基层体育基础设施建设，建成会宁县全民健身中心，推进平川区、景泰县体育中心建设，贫困村“一村一场”实现全覆盖。加快“信息惠民”试点工作，实现政务服务“一号一窗一网”目标，基本公共服务事项网上办理率达到80%以上。

真情为民，让社会保障更有力。实施一揽子措施促进就业创业，新增城镇就业5万人。完善城乡居民基本养老保险制度，续保率达到95%以上。整合城镇居民医保和新农合两项制度，进一步提高筹资水平。扩大城乡居民大病保险范围，提高报销比例。开展“敬老文明号”创建活动，做好城市社区居家养老服务和农村留守老人日间照料帮扶工作。认真落实残疾人两项补贴制度，加强对农村留守妇女、儿童的帮扶关爱。

真情安民，让社会发展更和谐。深化信访制度改革，健全多元化矛盾纠纷调解机制，畅通群众诉求表达渠道，妥善化解各类不稳定因素。加强农民工工资拖欠整治，构建和谐劳动关系。强化食品药品监管，厘清市、县、乡三级监管机构事权。做好社区矫正、人民调解、法律援助、法律服务、“七五”普法等工作，提高全社会法治意识。着力建立安全生产风险管控和隐患排查治理双重预防机制，杜绝重特大事故发生。完善立体化社会治安防控体系，严厉打击暴力恐怖、涉黑涉恶犯罪，切实维护人民群众生命财产安全。全面落实拥军优抚安置政策，巩固和发展军政军民团结局面。

集中力量兴办10件惠民实事:（1）新建市区过街天桥2座。（2）建设市、县区城区停车场60个。（3）为297个建档立卡贫困村每村新增扶贫互助资金配套5万元。（4）为已建成的农村幼儿园补充教师500名。（5）为全市400名一级肢体残疾少年儿童每人配备1辆电动轮椅。（6）设立农村互助老人幸福院运营奖补资金。（7）设立市级创业就业扶持专项资金，支持5000名大中专毕业生、失业人员、返乡农民工等创业就业。（8）建设“一村一场”农民体育健身工程100个。（9）实施棚户区改造8733户（套）。（10）完成农村危房改造1万户。

各位代表！新形势、新任务对政府工作提出了新的更高的要求，我们必须牢固树立“四个意识”特别是核心意识、看齐意识，以新的发展理念引领政府自身建设，着力推动政府治理体系和治理能力现代化，切实提升政府公信力和执行力。

强化法治思维。推动政府部门和工作人员自觉学法、守法、尊法、用法，厉行法治，依法行政，把政府工作全面纳入法治轨道。大力推进依法治市，全面落实法治政府建设实施纲要，在城乡建设与管理、环境保护、历史文化保护等领域制定地方性法规，加强行政执法监督，规范行政执法行为，完善行政执法人员以案释法制度和重大行政执法决定法制审核制度。自觉接受人大及其常委会依法监督和政协民主监督，提高人大代表建议和政协提案办理质量。全面推进政务、财政预算、公共资源交易等方面信息公开，完善新闻发布制度，充分利用微博、微信等新媒体，及时传递政务信息，回应社会关切。

强化改革意识。进一步解放思想，坚决破除传统思维定势和工作惯性，不断创新政府管理方式和工作方法。积极推行“互联网+政务服务”，从解决人民群众反映强烈的办事难、办事慢、办事繁等问题出发，简化优化办事流程，推进线上线下结合，提供渠道多样、简便易用的政务服务。全面推行“五证合一、一照一码”和“双随机、一公开”改革，健全随机抽查事项清单、检查对象名录库和执法检查人员名录库，加快构建事中事后监管体系。继续深化预算管理制度改革，加强政府债务使用和管理。

强化担当精神。采取有力措施，着力解决政府工作人员中存在的能力不足“不能为”、动力不足“不想为”、担当不足“不敢为”问题，大力弘扬踏石留印、抓铁有痕的工作作风和钉钉子精神，形成层层负责抓落实、人人作为谋发展的工作局面。推行“一线工作法”，以严实的作风推动问题在一线发现、一线研究、一线解决。大力开展“治庸、治懒、治散”活动，倡导马上办、主动办、透明办，做到有令则行、行则迅速，有禁则止、止则彻底。完善政府绩效评价和目标管理责任制，加强动态督查和全过程考核，做到不搪塞、不推诿、不拖拉，推动每项工作善始善终、每项政策落到实处。

强化廉政观念。把纪律和规矩挺在前面，严格执行《关于新形势下党内政治生活的若干准则》、《中国共产党党内监督条例》和廉洁自律准则、纪律处分条例等党纪党规，全面落实党风廉政建设主体责任和“一岗双责”。健全惩治和预防腐败体系，加强财政收支、政府投资、工程建设、精准扶贫、社会保障等重点领域的行政监察和审计监督，推动监察和审计全覆盖，从源头上遏制和预防腐败行为。加大违纪违法案件查办力度，严肃查处发生在群众身边的腐败问题，树立风清气正、廉洁高效的政府形象。

各位代表！新一届政府使命光荣，责任重大。让我们紧密团结在以习近平同志为核心的党中央周围，在省委、省政府和市委的坚强领导下，坚定信心，锐意进取，勇于创新，真抓实干，为全面建成小康社会、建设繁荣文明幸福新白银而努力奋斗！
